# The NagY antiterminator in *Enterococcus faecalis:* a novel regulatory mechanism and its impact on cell metabolism

**DOI:** 10.1128/spectrum.01137-25

**Published:** 2025-08-29

**Authors:** Diane Soussan, Marine Salze, Blandine Harel, Victor Combret, Erwan Bourdonnais, Isabelle Rincé, Sabrina Gueulle, Dimitri Fayolle, Alain Rincé, Cécile Muller

**Affiliations:** 1 Université Caen Normandie, Normandie Univ, CBSA 27003 , Caen, France; 2 Université Caen Normandie, Normandie Univ, CERMN, DruiD platform 27003 , Caen, France; University of Florida College of Dentistry, Gainesville, Florida, USA

**Keywords:** antiterminator, regulation, N-acetylglucosamine, ribonuclease, *Enterococcus faecalis*, metabolism

## Abstract

The *Enterococcus* genus is the most controversial group of lactic acid bacteria. While some strains are used as probiotics, other species, including *Enterococcus faecalis*, are responsible for health-related pathologies. Under conditions of infection, the N-acetylglucosamine metabolism of *E. faecalis* undergoes significant changes in expression, even more important than those of virulence factors. This metabolism is mediated by the *nagY-nagE* operon, which is regulated by the transcriptional antiterminator NagY. In this report, we focus on the regulatory mechanism of NagY and its impact on bacterial metabolism. We showed that NagY requires the interaction with the RNase III to achieve optimal induction of its own expression by cleaving the 5′ untranslated region of the *nagY* mRNA. The NagY regulon was identified and the central role of the antiterminator in the *E. faecalis* metabolism was demonstrated, highlighting its importance in the opportunistic nature of the bacterium. This study provides a valuable advance in the understanding of regulation and the importance of post-transcriptional actors in *E. faecalis* adaptation.

**IMPORTANCE:**

As a commensal*, Enterococcus faecalis* colonizes the gastrointestinal tract of 31 to 80% of the intestinal microbiota in adults and is considered ubiquitous, due to its strong environmental stress resistance capabilities. However, in immunocompromised patients, the poorly understood transition from commensal to opportunistic pathogen occurs, and many studies suggest that the metabolism plays a central role in this process. In this study, we focus on the regulator NagY, which is involved in the metabolism of N-acetylglucosamine, an important carbon source for bacterial pathogens in the human host. We characterized a novel regulatory mechanism involving the NagY antiterminator and the ribonuclease RNase III. In addition, we identified the target genes of the regulator, through which we were able to demonstrate that NagY has a strong impact on the metabolism of β-glucosides. Overall, this work highlights the importance of regulation of the bacterial metabolic adaptation in the host.

## INTRODUCTION

*Enterococcus faecalis* is a pathobiont considered a clinical pathogen of major importance responsible for healthcare-associated infections, including urinary tract infections and endocarditis. However, this microorganism lacks most of the virulence determinants found in pathogenic bacteria: its ability to colonize is due to its resistance to many stresses and its metabolic activities. Since 1970, enterococci have spread easily in healthcare facilities, presenting intrinsic and acquired antibiotic resistance, causing infections that are difficult to treat (1). According to antimicrobial resistance surveillance in Europe in 2023, an unchanged high level of gentamicin resistance and an increasing number of isolates (59.3% increase from 2017 to 2021) indicate that *E. faecalis* remains a major challenge in the prevention and control of nosocomial infections (2).

As a member of the lactic acid bacteria (LAB), *E. faecalis* is defined as a gram-positive, non-aerobic but aerotolerant, acid-tolerant, and strictly fermentative bacterium, with lactic acid as the major end product of sugar fermentation (3–5). This bacterium is a fastidious organism that requires rich and complex nutrients for growth, like carbohydrates, amino acids, vitamins, and minerals. LAB import and degrade carbohydrates and related compounds through various metabolic pathways to promote their growth. One of the preferred carbon sources found in the host by pathobionts is the N-acetylglucosamine (NAG), as it is found abundant in the extracellular matrix, mucus, or macrophages (6, 7). NAG has been shown to be imported through the MptBACD permease complex (which also transports glucose and mannose) and the EIICBA^NAG^ phosphotransferase system (PTS) transporter NagE (8). The PTS is a multiprotein phosphorelay that couples the transport of the carbon source across the cytoplasmic membrane with its simultaneous phosphorylation (9).

We have previously characterized the NagY antiterminator, which plays a central role in the NAG transport and metabolism in *E. faecalis* (10), following the well-known model of the BglG and SacY families model of *Escherichia coli* and *Bacillus subtilis*, respectively. This latter regulator controls the inducible expression of genes involved in sucrose utilization in *B. subtilis* (11), namely the *sacB* gene, encoding a levansucrase implicated in the degradation of sucrose, and the *sacXY* operon, encoding the sucrose-specific EIIBC^Suc^ (SacX) and SacY itself. In the presence of sucrose, SacY binds the *sacB*-initiated transcript and its own mRNA at the 5´ untranslated region (UTR), and more specifically at the ribonucleic antiterminator (RAT) sequence. This interaction promotes the antitermination hairpin, which prevents the closure of the terminator structure and allows *sacB* and *sacXY* transcription. Sucrose is then phosphorylated by SacX during its import into the cell and metabolized. In the absence of the specific carbohydrate, SacY is phosphorylated and inactivated by SacX (12–16). Like SacY, the regulation of the *nagY-nagE* bicistronic operon in *E. faecalis* is mediated by NagY itself, by binding to a specific RAT sequence at the 5′ UTR of the nascent mRNA (10).

A previous study showed that during an *E. faecalis* infection of mouse peritoneum, the genes encoding proteins involved in metabolism, carbohydrate transport (including NagE), and regulation undergo significant changes in expression, more than virulence factors *per se* (17). Expression regulation is an important feature in bacteria to adapt to their environment and to establish colonization, especially in opportunistic pathogens such as *E. faecalis*. In addition to regulators like NagY, which have a direct role in gene expression, ribonucleases (RNases) are essential in almost all aspects of RNA metabolism including RNA maturation, degradation and turnover, quality control, and even as mediators of regulation (18). The RNase III was described as the first specific double-stranded RNA (dsRNA) endoribonuclease in *E. coli* (19). More specifically, RNase III can degrade perfectly complementary dsRNA and secondary structures formed within the same RNA molecule such as hairpin structures (20). In *B. subtilis,* the RNase III activity has been demonstrated as a double strand-specific endoribonuclease on rRNA and mRNA (21, 22). This enzyme is responsible for the maturation of tRNA precursors and rRNA or the degradation of regulatory RNA-mRNA duplexes, with consequences on cellular adaptation to stress (23, 24). In *E. faecalis*, the RNase III is a 26.2 kDa protein encoded by the *rnc* gene. This enzyme acts in dimer form and has an impact on the adaptive abilities of the bacteria, like cold-shock, oxidative, bile salt stress response, and even pathogenicity (24).

In this work, we have demonstrated a novel co-regulation mechanism involving NagY and the RNase III on the *E. faecalis nagY-nagE* operon. In parallel, we aimed to characterize the NagY regulon using global approaches. These analyses allowed us to distinguish specific patterns of deregulation attributed to RNase III and highlighted common regulatory pathways mediated by both NagY and RNase III, leading to a better understanding of their coordinated role in gene regulation. The identification of the NagY regulon also revealed that the antiterminator plays a central role in the utilization of carbon sources found in the host. This opens up new perspectives for understanding infections caused by opportunistic pathogens like *E. faecalis*.

## RESULTS

### Identification of a new regulation mechanism of the *nagY-nagE* operon by NagY

**Fig 1:**
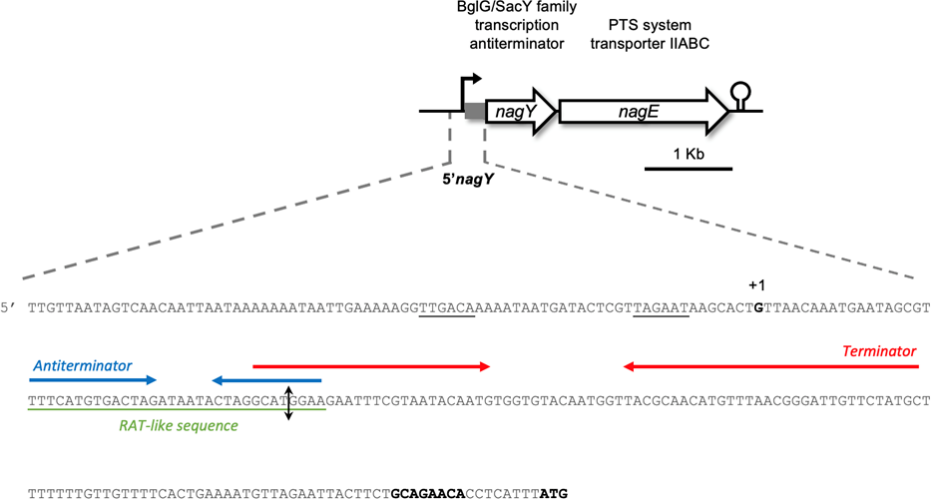
Scheme of the *nagY-nagE* operon and its 5′ untranslated region. In the 5′ UTR (5′ *nagY*, upper panel), the transcription start site identified in this study (+1, G), the ribosome binding site (GCAGAACA), and the translation start site (ATG) are indicated in bold, the −10 and −35 boxes are underlined. The RAT-like sequence is underlined in green, and the identified terminator and antiterminator are overlined by inverted arrows in red and blue, respectively. The identified RNase III cleavage site is indicated by a double black arrow.

In a previous study, we reported that the *nagY-nagE* operon in *E. faecalis* is regulated by the NagY antiterminator, following the canonical SacY model, in which the terminator hairpin overlaps the RAT sequence, i.e., the antiterminator binding site (Fig. 1) (10). The NagY binding allows the formation of the antitermination loop and the expression of the *nagY-nagE* operon in the presence of NAG in the culture medium.

**Fig 2:**
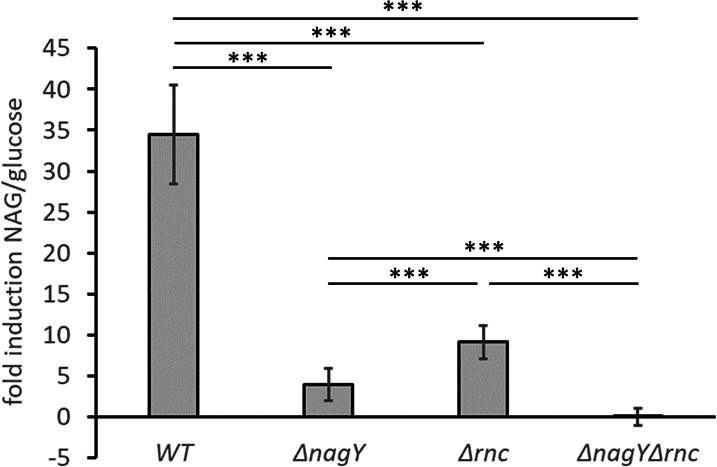
Fold change of *nagE* gene expression in the presence of NAG compared to glucose condition. RT-qPCR was performed on RNA extracted from *E. faecalis* V19 (WT), Δ*nagY*, Δ*rnc,* and Δ*nagY*Δ*rnc* strains in exponential phase and then exposed to glucose or NAG as sole carbon source for 1 h. Error bars represent data from triplicate independent experiments, and asterisks indicate statistically significant differences determined by the Tukey multiple comparison test, ***P < 0.0001.

To monitor the expression of the *nagY-nagE* operon, RT-qPCR assays targeting *nagE* were carried out on RNA extracted from the ∆*nagY* and wild-type (WT) strains grown in carbon-depleted cdM17 media supplemented with glucose until exponential growth phase and exposed to NAG or glucose (control) as carbon source for 1 h to induce or not NagY expression, respectively. As shown in Fig. 2 and as expected, the *nagE* expression is induced more than 35-fold in the presence of NAG compared to the glucose condition in the WT strain. However, when the *nagY* gene is deleted, the transcription level is reduced, but not completely abolished with a fivefold induction, suggesting the existence of another regulatory mechanism.

To determine whether this residual expression is dependent on the *nagY* untranslated region or not, the previously constructed mutant deleted for the untranslated region sequence 5′ *nagY* was used (10). In this strain, *nagE* expression is always induced regardless of the carbon source (fold change [FC]: NAG/glucose = 1.3 ± .2; data not shown), suggesting that the entire carbohydrate-dependent regulation involves intrinsic element(s) on 5′ *nagY*.

**Fig 3:**
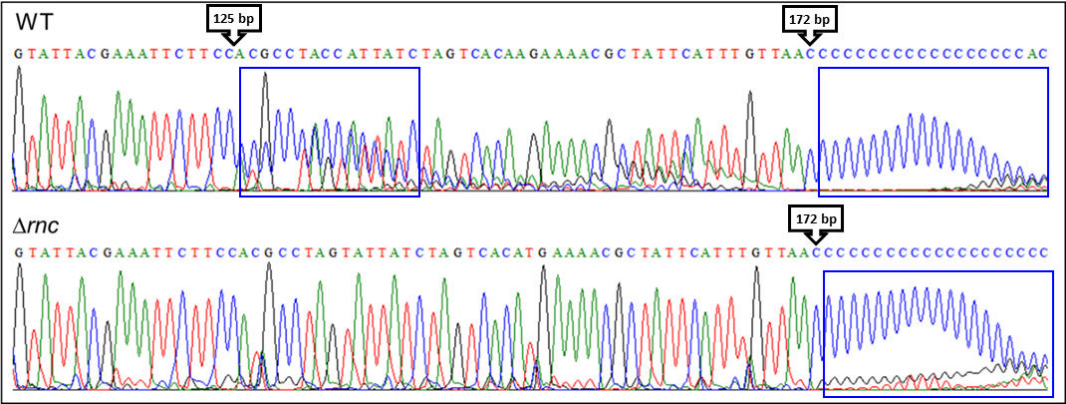
Electropherogram of the 5′ RACE-PCR sequence performed on 5′ *nagY*, in the WT strain (top panel) and the Δ*rnc* strain (bottom panel), with a poly-C tail. The arrows indicate the nucleotides, and their position compared to the ATG, on which the poly-C tail (framed in blue) is anchored, and thus the complementary bases of the 5′ ends of the RNA.

To better understand this regulation, the transcription start site was identified by 5′ rapid amplification of cDNA ends-PCR (RACE-PCR), using poly-C tailing. As shown in Fig. 3, the start base of the RNA was located 172 bp before the predicted initiation codon, but another RNA extremity was also observed 125 bp before the ATG (represented by the vertical double arrow in Fig. 1). This result can be explained by two hypotheses: (i) there are two independent promoters allowing two independent initiation start sites, and (ii) the transcript is matured by a ribonuclease. We have excluded the first hypothesis because no other −10 and −35 boxes were identified on the 5′ *nagY* sequence.

Double strands in mRNA can be specifically recognized and hydrolyzed by RNase III. Since the 5′ *nagY* RAT sequence has been shown to form a hairpin structure, we hypothesized that this degradative enzyme recognizes and cleaves this stem-loop. We used a previously constructed mutant deleted in the RNase III encoding gene *rnc* (24) and performed 5′ RACE-PCR experiments on RNA extracted from this strain. When the bacterium was deprived of RNase III, no cleavage site was detected (Fig. 3), in contrast to the WT strain, confirming that RNase III is involved in the rift of the conserved secondary structure in the 5′ UTR.

The cleavage site is located on a sequence common to the antiterminator and the terminator stem-loops: RNase III-mediated RNA maturation could therefore interfere with the NAG-dependent regulation of the *nagY-nagE* operon. However, when *nagE* expression is determined by RT-qPCR on RNA extracted from the Δ*rnc* mutant (Fig. 2), we observe that *nagE* expression is still induced when we compare NAG *vs* glucose culture conditions. In the Δ*nagY*Δ*rnc* double mutant (constructed as described in Materials and Methods), the expression induction of the *nagY-nagE* operon is completely abolished (Fig. 2). This demonstrates that NagY cooperates with the RNase III to carry out the NAG-dependent induction of its own operon.

### Interaction of RNase III with 5′ nagY and/or NagY *in vitro*

**Fig 4:**
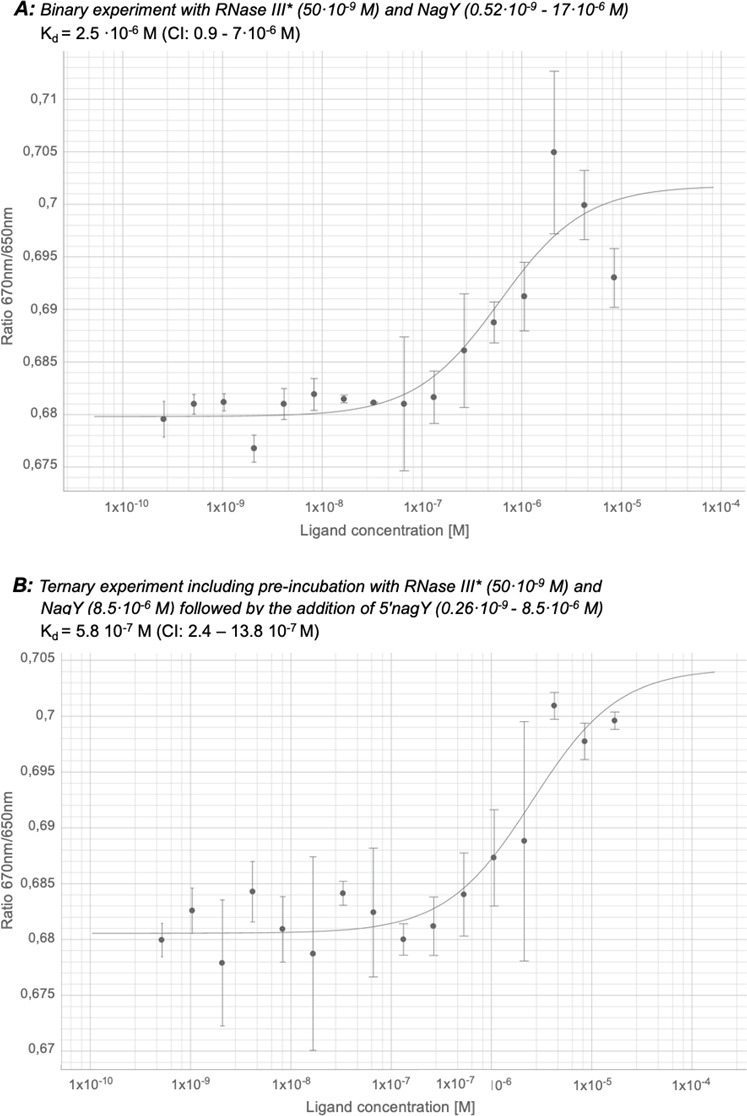
Investigation of the interaction of RNase III-labeled (RNase III*) with NagY and/or its 5′ UTR. Spectral shift (OD 670_nm_/650_nm_) is shown for the interaction between (**A**) RNase III* (50·10^−9^ M) and NagY (0.52·10^−9^–17·10^−6^ M) and (**B**) RNase III* (50·10^−9^ M) and NagY (8.5·10^−6^ M) followed by the addition of 5′ *nagY* (0.26·10^−9^–8.5·10^−6^ M). Figures A and B refer to assays 2 and 4; see Table S1 and Fig. S1 for tests 1, 3, and 5. Error bars represent data from triplicate independent experiments.

To confirm that the transcriptional antitermination of *nagY-nagE* is regulated by the RNase III, the direct interaction between this protein and 5′ *nagY* RNA and/or NagY was examined by spectral shift of fluorescence-labeled RNase III (RNase III*) (Table S1). The RNase III was purified, cysteine-labeled (degree of labeling [DOL] 0.1 fluorescent tags per protein unit), and first incubated in the presence of *in vitro* produced 5*′ nagY* RNA, but no clear interaction was observed (Fig. S1). In contrast, a dose-response curve indicated the binding of RNase III* to NagY, with a K_d_ of 2.5 ·10^−6^ M (confidence interval [CI] = 0.9–7·10^−6^ M) (Fig. 4A).

The three-partner interactions between the RNase III, NagY, and 5′ *nagY* were also monitored and showed that the choice of the first two partners brought into contact has an impact on the interaction and affinity. Indeed, when the RNase III* was first mixed with the NagY protein and then with the 5′ *nagY* RNA, an interaction of the RNase III* on RNA with a K_d_ of 5.810–7 M (CI = 2.4–13.8 10^−7^ M) was measured (Fig. 4B). In contrast, when the labeled ribonuclease was added after NagY and 5′ *nagY*, or NagY after the RNase III*-RNA pair (Fig. S1B and C, respectively), no interaction was observed. These results suggest that an initial interaction between NagY and 5′ *nagY* prevents the involvement of the RNase III protein *in vitro*, while the formation of the NagY-RNase III complex increases the affinity of the ribonuclease for 5′ *nagY* RNA.

### The NagY antiterminator and the RNase III are both involved in *nagY-nagE* regulation *in vivo*

**Fig 5:**
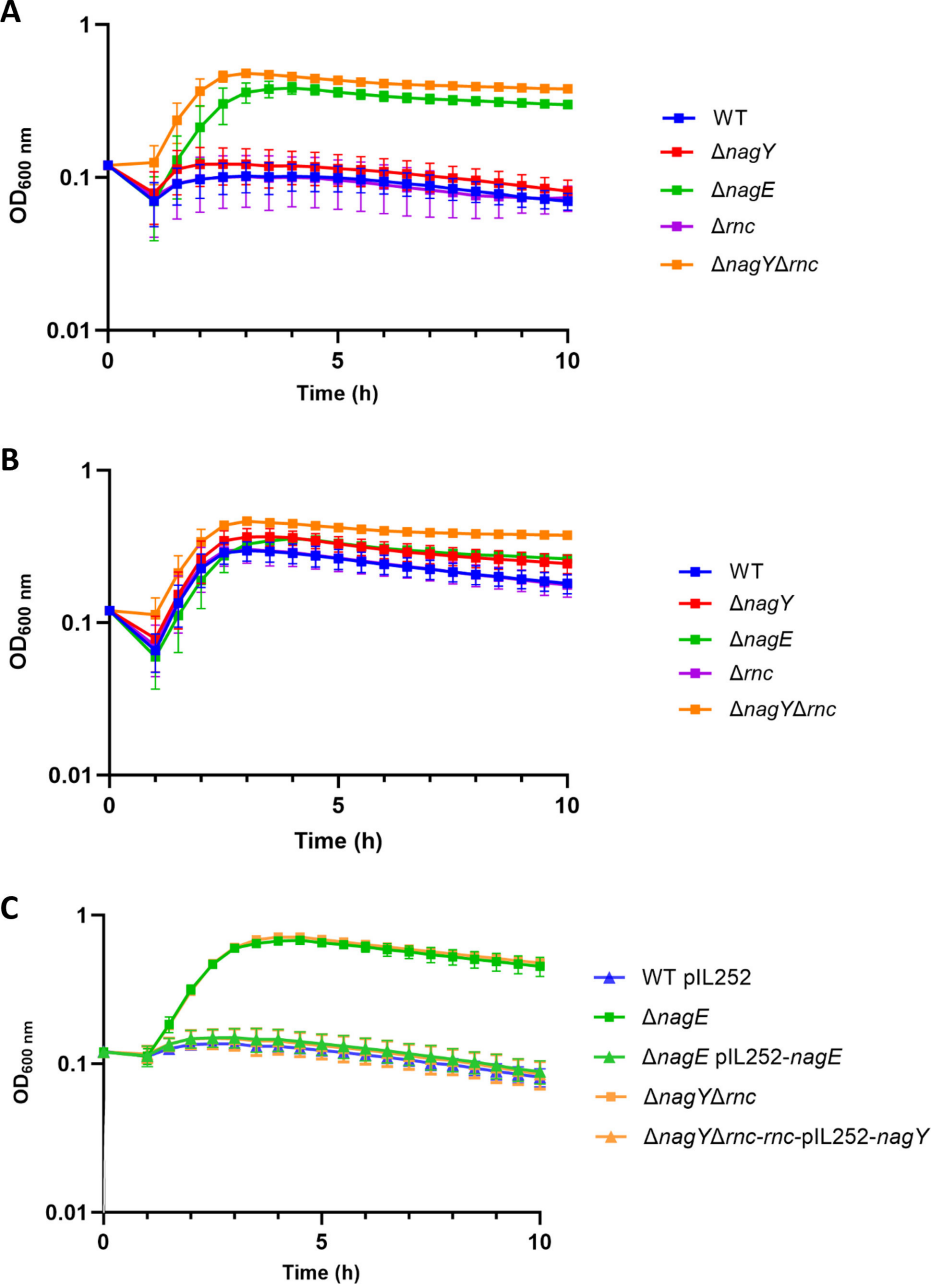
Growth in the presence of STZ. Growth of *E. faecalis* mutant strains was monitored in GM17 and in the presence of 50 µg·mL^−1^ STZ (**A**) or 50 µg·mL^−1^ STZ and 0.5% NAG (**B**), and Δ*nagE* and Δ*nagY*Δ*rnc* with their complemented strains in the presence of 50 µg·mL^−1^ STZ (C). Overnight cultures were performed in GM17 supplemented with 0.5% NAG. Error bars represent data from three independent experiments, and data were analyzed with the Tukey multiple comparison test.

To confirm that the RNase III is involved in the expression of the *nagY-nagE* operon, we used Δ*nagE* (constructed as described in Materials and Methods), ∆*rnc,* ∆*nagY,* and Δ*rnc*Δ*nagY* mutant strains to perform physiological studies (10, 24). We first checked that all strains had similar growth rates under our culture condition (Fig. S2). Considering that *E. faecalis* can import NAG through the NagE PTS but also through the Mpt glucose/mannose permease complex system (8), we examined the growth of Δ*rnc,* Δ*nagY*, Δ*nagE,* and Δ*rnc*Δ*nagY* mutants in the presence of streptozotocin (STZ), a bacteriostatic antibiotic that has been reported to be specifically transported through the PTS^NAG^ in *Streptococcus mutans* (25). As shown in Fig. 5A, an inhibitory effect of STZ on WT growth was observed but not for the Δ*nagE* mutant (*P* < 0.0001), confirming the transport of STZ by NagE. To determine whether the Mpt transporter played a role in this transport, a complementary study of the expression level of the *mptBACD* transcript was carried out (Fig. S3). No induction of the Mpt transporter was measured in our culture condition in the presence of NAG compared to glucose (*P* < 0.0001).

Furthermore, when NAG was constantly present in the culture media (GM17 supplemented with 50 µg.mL^−1^ STZ and 0.5% NAG), STZ did not inhibit the growth of WT, confirming that STZ and NAG compete for the same transporter (Fig. 5B). The ∆*rnc* or ∆*nagY* mutants had a similar change in growth as the WT strain in the presence of STZ (Fig. 5A), indicating that STZ was still transported by NagE and exerted its inhibitory effect. This result was consistent with that of RT-qPCR assays where a residual expression/induction of *nagE* was detected in both single mutants (Fig. 2). In contrast, STZ had no effect on the ∆*nagY*∆*rnc* mutant compared to the other strains (*P* < 0.0001), and as observed in the Δ*nagE* mutant (Fig. 5A), suggesting that NagE is not expressed.

We also determined the minimal inhibitory concentrations (MIC) of STZ against all strains (Table 1). The WT, Δ*nagY,* and Δ*rnc* strains exhibited similar susceptibility to STZ with an MIC ranging from 25 to 50 µg/mL. One possible hypothesis is that the slight difference in MIC between the Δ*nagY* and Δ*rnc* mutants is due to the difference in *nagE* expression observed in Fig. 2. By contrast, the Δ*nagE* and Δ*nagY*Δ*rnc* mutants were both much more resistant to STZ, with an MIC of 800 µg/mL, which is consistent with the data in Fig. 5A.

**TABLE 1:** MIC of STZ for the individual *E. faecalis* strains used in this study

Strains	MIC (µg/mL)
WT	25
Δ*nagY*	50
Δ*nagE*	800
Δ*rnc*	25
Δ*nagY*Δ*rnc*	800
WT pIL252	25
Δ*nagE-*pIL252-*nagE*	50
Δ*nagY*Δ*rnc-rnc-*pIL252-*nagY*	50

Complemented strains of the ∆*nagY*∆*rnc* and ∆*nagE* mutants were constructed as described in Materials and Methods. These strains were then used to perform the STZ inhibition assays to eliminate the involvement of mutations in non-target genes. As shown in Fig. 5C and Table 1, the complemented strains present the same phenotype as the WT strain, in the presence or absence of STZ, confirming that the ∆*nagY*∆*rnc* and ∆*nagE* phenotypes are exclusively due to those mutations.

Taken together, these results confirm that NagY and RNase III are both involved in co-regulating the expression of the *nagY-nagE* operon. While only one of these two partners is required for a basal NAG transport, both must be present to ensure efficient import of NAG.

### Identification of new NagY antiterminator targets

We have previously reported that NagY was able to regulate the expression of its own operon *nagY-nagE* and the *hylA* gene*,* which encodes a polysaccharide lyase involved in glycosaminoglycan degradation (10). Thus, NagY seems to play a pleiotropic role in metabolism, and we performed a transcriptomic study to identify novel targets for NagY. To this end, RNA-Seq was carried out using the WT and ∆*nagY E. faecalis* strains cultivated in GM17 until exponential growth phase, followed by an induction of 1 h in the presence of NAG as sole carbon source. After filtering low-quality reads, approximately 30.10^6^ reads were obtained, of which 84 to 90% were uniquely mapped to the reference genome (AE016830.1) for the mutant and WT strains, respectively (Table S2).

We arbitrarily chose to examine genes with an FC in expression of 5 and above when comparing mutant and WT strains, and these differentially expressed genes were classified using the UniProt database (https://www.uniprot.org/) (Table 2; Table S2 and Table S3 for details).

**TABLE 2:** Gene ontology classification (biological process in prokaryotes, gathered by function categories) of induced and repressed genes in the Δ*nagY*, Δ*rnc,* and Δ*nagY*Δ*rnc* strains compared to the WT condition (FC ≥ 5)

GO annotations	Δ*nagY*		Δ*rnc*		Δ*nagY*Δ*rnc*	
	Induced	Repressed	Induced	Repressed	Induced	Repressed
Transport	1	27	1	8	1	30
Metabolism process	3	29	2	15	1	36
Gene expression	4	9	10	4	13	5
Cell organization and signaling	1	9	1	1	0	11
Hypothetical or unclassified proteins	11	10	6	4	10	13
Total	104		52		120	

A total of 104 genes were differentially expressed in the ∆*nagY* mutant compared to that in the WT strain, most of which are repressed (84 repressed vs 20 induced), consistent with the antiterminator role of NagY. These results show that NagY has an inductive and repressive effect in *E. faecalis*. The classification shown in Table 2 highlights that most of the repressed genes are involved in metabolism (29 genes) and transport (27 genes), with 16 PTS-type transporters, including *nagE* (FC = −14.4-fold), and 7 ABC transporters. In the metabolism class, deregulated genes are involved in anabolic and catabolic processes, including the previously identified NagY target *hylA* (FC = −69.8-fold).

This transcriptomic study should be cautioned by the fact that all the deregulated genes, i.e., direct and indirect targets of NagY, emerged between the two strains. Therefore, we used RNA immunoprecipitation sequencing (RIP-Seq), which allows the study of *in vivo* interaction between NagY and specific RNA (26, 27). The NagY protein was first tagged with a FLAG peptide and expressed in *E. faecalis* WT and then used to capture target RNA by co-immunoprecipitation (co-IP).

We first verified that the proteins were stably expressed and detectable in the lysate (Supplemental method and Fig. S4). The *E. faecalis* strain expressing the NagY protein with a FLAG epitope and the WT strain (negative control) were then cultured under the same conditions as used for the previous transcriptomic study. After co-IP, the bound RNAs were purified and sequenced by RNA-Seq. The RIP-Seq analysis was based on genes with more than five reads that were detected only in the WT strain harboring the NagY-FLAG protein (Table S4). Genes were then classified using the UniProt database (https://www.uniprot.org/). The results are presented in Table S5.

In the control strain (without the NagY-FLAG proteins), only a few genes emerge from the analysis with less than five reads (*n* = 18), showing that the recombinant protein binds its targets with high specificity. In total, 57 RNAs co-precipitated with the NagY-FLAG protein in our culture conditions, including 27 common to the RNA-Seq assay and the *nagY-nagE* and *hylA* targets. We observed that no small non-coding RNA co-immunoprecipitated with the tagged protein. Most of the genes correspond to the first genes of operons, which is consistent with the technology used.

**Fig 6:**
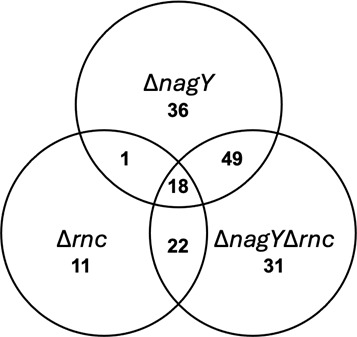
Deregulated genes common to the transcriptomes of *E. faecalis* Δ*nagY*, Δ*rnc,* and Δ*nagY*Δ*rnc. E. faecalis* strains were grown in GM17 until exponential growth phase, followed by induction for 1 h in the presence of NAG as sole carbon source. The reads were mapped to the reference genome AE016830.1.

Since the NagY antiterminator interacts with the RNase III (K_d_ of 2.5 ·10^−6^ M, Fig. 4), it can also co-precipitate with this partner and RNAs being degraded/matured by the ribonuclease. To eliminate RNAs that are not direct targets of NagY, a transcriptomic study was also performed using the *E. faecalis* ∆*rnc* strain and the ∆*nagY*∆*rnc* double mutant to identify co-regulated genes (Table 2; Table S2; Table S3). A total of 120 genes were identified as deregulated in the ∆*nagY*∆*rnc* mutant, compared to 104 and 52 genes in the ∆*nagY* and ∆*rnc* single mutants, respectively (Fig. 6).

As attended, the ∆*rnc* strain transcriptome showed that known RNase III target genes in *Staphylococcus aureus* or *E. coli* are deregulated, such as *secY* (26, 28); *adhE*, *nusA*, *dnaK*, *pflB*, *ahpC,* or *ssrA* (29); *hup* and *groES* (26); and *eno, rnc,* and rRNA (26, 29). In the *E. faecalis* ∆*nagY*∆*rnc* strain, most of the direct and indirect targets of Nags are repressed (79%), especially in the metabolism and transport categories (Table 2 and Table S3).

**Fig 7:**
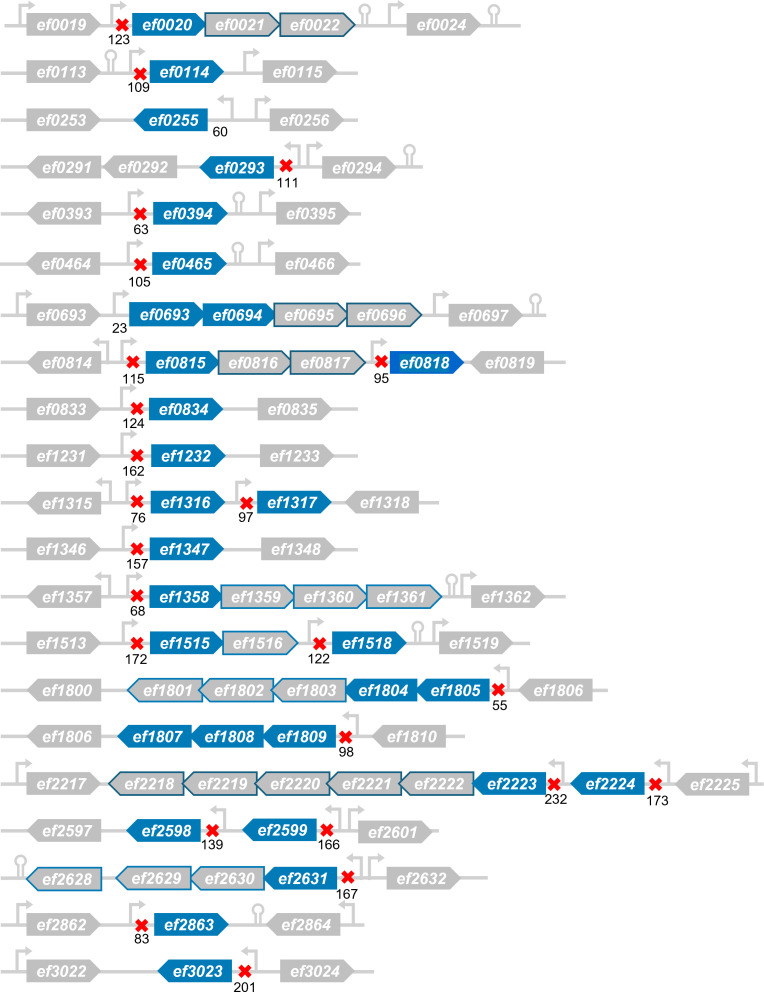
Genomic environment of the 26 direct targets of the NagY antiterminator identified by transcriptomic and co-immunoprecipitation analyses. Direct targets of the antiterminator confirmed by RIP-Seq are shown in blue, with the genes of the same operonic structure circled in blue. Transcripts +1 and terminators are shown according to previous global studies (17, 30, 31). Putative RAT sequences identified by the FIMO tool (32) with a *P*-value <0.01, within 200 bp upstream of the target genes, are represented by red crosses. Annotations are not to scale for better visibility.

In order to focus on the objective of this study, which is to identify the mechanism of NagY regulation and its regulon, we are only interested in the genes directly regulated by NagY among those identified by co-IP, leaving aside the RNase III targets. We conserved the genes whose expression was repressed in the ∆*nagY* mutant compared to the WT (Table 2). These genes and their operons are represented in Fig. 7.

A majority of these genes (in operon or not) are involved in sugar transport or metabolism. For example, 15 genes encode PTS subunits annotated as belonging to mannose/fructose (*ef0020-ef0021-ef0022, ef0694-ef0695, ef0815-ef0816-ef0817, ef1804-ef1803-ef1802-ef1801*), cellobiose (*ef0292* and *ef0834*), or β-glucoside (*ef2598*) transporter family, and four encode ABC sugar transporter subunits (*ef1232* and *ef2223-ef2222-ef2221*). In terms of metabolism, NagY target gene products are involved in the hydrolysis of large glycoproteins or polysaccharides, such as glycosyl hydrolases (*ef0114, ef0291, ef1347, ef1805, ef2863*) or polysaccharide lyases (*ef0818* and *ef3023*), as well as primary metabolism, like those of NAG (*ef1317*), glycerol (*ef2358-ef1359-ef1360-ef1361*), tagatose (*ef0693*, *ef0696*, *ef1808-ef1807-ef1806*), or lactate (*ef0255*). Sensors and regulators are also represented (*ef0293, ef1809, ef2219-ef2218*), as well as secreted or cell wall-anchored antigen (*ef0394* and *ef2224*) and the operon *ef2631-ef2630-ef2629-ef2628* whose function is unknown.

We also searched for the RAT sequence homologous to that found in the 5′ *nagY* sequence (Fig. 1) in the 200 bp before the ATG of each identified target. Of the 26 direct targets identified, 23 genes have a putative RAT sequence (Table S5, represented by a red cross in Fig. 7), strongly suggesting that they belong to the NagY regulon.

## DISCUSSION

*E. faecalis* is a bacterium capable of causing healthcare-associated infections due to its ability to survive outside and inside the host and to adapt its metabolism (1). This link between metabolism and virulence has recently been highlighted in *E. faecalis* by the study of the antiterminator NagY (10). This protein induces the expression of a hyaluronidase involved in biofilm maintenance, infection, and degradation of hyaluronic acid, a major component of the host extracellular matrix and the mucus. This degradation is thought to facilitate the bacterial proliferation by providing a source of carbon and energy. The NagY protein is capable of autoregulation due to its role in the transcriptional antitermination at the 5′ UTR of its own operon *nagY-nagE*.

### The RNase III-NagY co-regulation

Interestingly, another level of regulation of the *nagY-nagE* operon involving 5′ *nagY* was highlighted in this study. While NagY regulates the operon expression by binding an antiterminator sequence, the second level of regulation identified induces the formation of a 47 bp shorter transcript. The corresponding 5′ end of this mRNA is located in the hairpin structure formed by the previously identified RAT sequence overlapping the terminator sequence (10). The cleavage site at the stem-loop structure, and thus the double-stranded RNA, was shown to be RNase III dependent at the molecular level and *in vivo* (Fig. 3 and 5, respectively). It should be noted that RNase III is involved in the degradation of double-stranded RNA in *B. subtilis* (22). Furthermore, the stability of the 5′ *sacXY* mRNA has been suggested to imply a ribonuclease. Our results in *E. faecalis* suggest that the RNase III is this unknown actor and thus open up new research perspectives in *B. subtilis* or other model bacteria on the RNA-binding antiterminator mechanism.

We have shown that NagY and the RNase III are complementary to achieve a full induction of the *nagY-nagE* operon. However, the interaction between RNase III and 5′ *nagY* was not detected *in vitro,* which could be explained by the intervention of another partner *in vivo*. For example, the optimal expression of the *sacXY* operon and the *sacB* gene in *B. subtilis* is enabled by the intervention of two regulators: the SacY antiterminator and the DegU protein of the two-component DegS-DegU regulatory system. The signal transmitted by the positive regulator DegU and the presence of sucrose represent the conditions under which levan synthesis by levan-saccharase is useful for the cell (15, 33).

We have shown that the interaction of the RNase III, NagY, and 5′ *nagY* RNA has a higher affinity than the two-partner RNase III-NagY interaction *in vitro* and that the RNA has to be added after the NagY-RNase III interaction. The lack of interaction in the three-partner assay between RNase III and 5′ *nagY,* followed by NagY, is probably due to a stronger affinity interaction between NagY and 5′ *nagY in vitro* (10). These observations suggest that NagY is the central sensor of the presence of NAG, which conditions the intervention of the RNase III.

To our knowledge, the interaction between the NagY regulator and the RNase III is the first evidence of a couple of proteins involved in a post-transcriptional antiterminator mechanism in bacteria. The RNase III has previously been shown to be involved in complex mechanisms involving three partners. The RsaE RNA, which is implicated in the production of cytolytic toxins in *S. aureus*, is negatively regulated by the binding of the RsaI RNA and the cleavage of the duplex formed by RNase III (34). In *E. faecalis,* the conjugation ability is mediated by the *prgQ* operon, which is negatively regulated by the anti-Q RNA and the transcriptional repressor PrgX. The *pgrX* mRNA is then targeted by a Qs RNA to form a complex that is then cleaved by RNase III. This ribonuclease thus limits the activity of the PrgX repressor and promotes conjugation (35). Both mechanisms described involve the ribonuclease and small RNA partners, but not RNA-binding proteins.

### The RNase III role

*E. faecalis* RNase III has already been shown to be involved in the stress response and the pathogenicity of the bacterium (24). Through the control of NagY, this enzyme is also indirectly involved in NAG metabolism, biofilm formation, and hyaluronic acid degradation (10). Other studies confirm that mutation of the *rnc* gene has an impact on biofilm formation and resistance to antibacterial agents in *E. faecalis* (36), as well as in *Salmonella* (37), or the expression of factors implicated in cell adhesion and escape from immunity (38). Moreover, in *E. coli*, RNase III has been suggested to have a strong effect on metabolism (39) and is considered a stress response modulator (23). This enzyme has consequently a pleiotropic role in gram-positive and gram-negative bacteria, with strong impact on virulence and metabolism (40).

Our transcriptomic approach of the *E. faecalis* Δ*rnc* mutant shows no significant change in transcript abundance, with 52 open reading frames deregulated at an FC >5. Several studies have focused on the RNase III regulon, and results can vary widely depending on the species or culture conditions. In *Streptococcus pyogenes,* RNase III does not have a strong effect on RNA abundance but can still control gene expression by affecting translation, with a preferential cut site in the UTRs (28), or by stabilizing/maturing the transcript (20). In *B. subtilis*, the RNase III depletion can control approximately 11% of total transcripts (41). In *S. aureus,* the RNase III gene deletion has a strong effect on the transcriptome, especially on the short RNA fraction (42), while little mRNA is recovered by co-immunoprecipitation (27). In these studies, it was observed that the majority of ribonuclease-regulated RNA is mainly non-coding RNA (small RNA and UTR), which could explain the low number of deregulated genes in the Δ*rnc* mutant under the specific condition of NAG induction. Thus, the RNase III affects NagY expression by cleaving the UTR 5′ *nagY* and could play a role in RNA stabilization and/or could prevent formation of the terminator structure. Since we only searched for NagY target genes, we did not analyze the RNase III regulon, which will be the subject of a future study.

### The NagY regulon

We used a dual approach to identify the NagY regulon. The RNA-Seq comparing the Δ*nagY* mutant and the wild-type strain allowed the identification of all the deregulated genes, whether direct targets or not. The RIP-Seq selected the mRNAs in direct interaction with the antiterminator and, consequently, the direct target genes. These experiments are complementary in the study of the RNA-binding protein regulon.

The co-IP of NagY with its target RNAs allows the recovery of the RNase III identified targets, such as rRNA, *ssrA*, *secY*, or *eno*, confirming the RNase III-NagY interaction *in vivo*. We chose to focus on repressed genes in the Δ*nagY* mutant for which their RNA interacts with the antiterminator. In total, 26 operon or monocistronic genes emerged from the study, including 23 with a putative RAT sequence in their 5′ UTR (Fig. 7).

It should be noted that although antitermination is the primary known function of antiterminators, they may have additional roles. For example, in *E. coli*, the BglG antiterminator has been shown to regulate its own operon *bglG-bglF*, but also at least 12 target genes that may have RAT motifs that are not associated with a transcription termination structure (43). In *B. subtilis*, 11 putative RAT sequences have been identified, in addition to those already known. As shown for *E. coli*, most of the putative RAT sequences did not overlap with terminators, suggesting roles other than antitermination (44).

Some of the mRNAs co-precipitated with NagY also lack homologous RAT sequences (Fig. 7 and Table S5): their NagY-dependent expression could involve another actor, such as the aforementioned protein DegU (15, 33), or an unknown mechanism yet to be identified.

**Fig 8:**
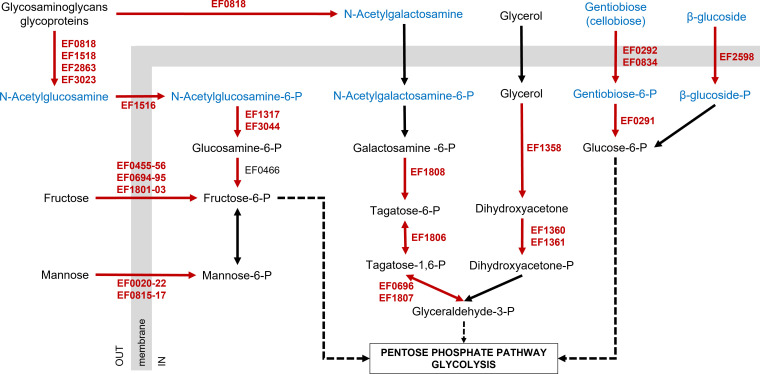
Schematic representation of the role of NagY targets in metabolism. Direct NagY targets identified in this study are shown in red, indirect targets in black, and β-glucosides in blue. P, phosphate.

Most of the NagY targets identified are involved in the transport of carbon sources and their metabolism, as shown in Fig. 8. It can be clearly seen that β-glucosides are the preferred carbon source, which is converted to fructose-6-P, glucose-6-P, or glyceraldehyde-3-P, subsequently translocated to glycolysis or pentose phosphate pathways. β-glucoside metabolism has been implicated in numerous mechanisms in pathogens like streptococci, including colonization, virulence, and biofilm formation (45, 46). As noted, NagY is a central regulator of the NAG utilization. In addition to its role in transport induction, it activates the expression of EF0465 and EF1316 proteins, two regulators of the *nagB* (*ef0466*) and *nag_A1* (*ef1317*) genes, respectively, both involved in the conversion of NAG to fructose. Previous work showed that gentiobiose transport and metabolism are strongly induced in *E. faecalis* during infection and are dependent on the GenR (EF0293) regulator (17, 47). Interestingly, the *genR* gene expression is NagY dependent, and consequently, the *ef0291-ef0292* operon, too. In parallel, the tagatose is probably produced from N-acetylgalactosamine. It should be noted that NAG, N-acetylgalactosamine, and mannose have been identified as central carbon sources for pathogen colonization (6, 7). Glycerol metabolism has been extensively studied in *E. faecalis* and has also been shown to be an important pathway for its colonization abilities, especially the *dhaK* (*ef1360*) pathway. All of these metabolic pathways have been shown to have an important impact on the pathogenicity of *E. faecalis*, for which the NagY regulator is the central denominator.

Among the NagY targets, we also identified four genes encoding glycosyl hydrolases. These enzymes cleave specific glycan linkages, with N-acetylglucosaminidase (GH20 family, EF0114 and GH18 family, EF2863), α-glucosidase (GH13 family, EF1347), or galactosidase (GH35 family, EF1805) activities. All of these proteins are thought to degrade host glycoproteins, containing short, highly branched N-, C-, or O-glycan chains with no repeating unit (48, 49). In the host, glycosylation has functions in protein folding, maturation, trafficking, or secretion, as well as in the immune system or in mucus composition. At the bacterial level, glycoproteins are indispensable components of the cell wall and facilitate adhesion and biofilm formation with implications for antibiotic resistance and can be used as a carbon source (50–55). In *E. faecalis*, glycoprotein-derived compounds affect pathogenesis by reducing bacterial virulence (56). Glycosaminoglycans found in the extracellular matrix are also an interesting saccharide source during bacterial colonization. They are composed of disaccharide repeats formed by NAG or N-acetylgalactosamine linked to uronic acid or galactose. Enzymes such as polysaccharide lyases (EF0818 and EF3023) are specialized for the degradation of glycosaminoglycans (57, 58). The NagY-dependent expression of these enzymes makes sense because they allow the recovery of easily utilizable metabolites (NAG or N-acetylgalactosamine). Pathobionts like *E. faecalis* are in constant contact with glycans throughout their lives, both as commensals and as opportunistic pathogens. Thus, these enzymes involved in glycoprotein metabolism represent potential targets for novel innovative therapeutic approaches, underscoring the importance of the NagY regulator.

The *ef1518* gene just downstream of the *ef1515-ef1516* operon (*nagY-nagE*) was also shown to be regulated by NagY. This gene encodes a cell wall hydrolase with an N-terminal lysozyme domain that cleaves the bond between N-acetylmuramic acid and NAG in peptidoglycan. This suggests that *E. faecalis* can recover NAG from bacterial peptidoglycan: investigation of the role of this protein will be necessary to confirm this hypothesis. The EF0394 or SalB secreted protein is required for stress response, antibiotic resistance, and maintenance of cell integrity (59, 60). The role of this protein as well as the cell wall-anchored protein EF2224 in the NagY regulon remains to be determined, but both have been shown to be expressed at higher levels during the infection process and to be highly immunogenic (61, 62).

Finally, we observed that NagY regulates its own homologous gene of the *E. faecalis* genome, *bglG* (*ef2599*), and *bglF* (*ef2598*), encoding its associated transporter, suggesting a link between the two regulons. The BglG-BglF associated system has not been studied to our knowledge, and this link will be the subject of future investigations into co-regulation and impact on the physiology and pathogenicity of the bacterium.

### Conclusion

The identification of the NagY regulon demonstrates the importance of this regulator in the β-glucoside metabolism and its potential impact on host interaction. This work provides a valuable overview of the connection between post-transcriptional regulators (i.e., transcriptional antiterminator and ribonuclease) and the importance of metabolism in pathobiont opportunism, opening new perspectives in the research of strategies to control infections.

## MATERIALS AND METHODS

### Bacterial strains and growth conditions

The strains used in this study are listed in Table 3. The *E. faecalis* V19 strain (WT) derived from the clinical strain V583 but cured from its plasmids was used as the reference strain (63, 64). *E. faecalis* was grown at 37°C in M17 medium supplemented with 0.5% glucose (GM17) or NAG. *E. coli* strains TOP10 (Thermo Fisher, Waltham, MA, USA), NEB-5α (New England BioLabs, Ipswich, MA, USA), DH-5α (Life Technologies, Carlsbad, CA, USA), and M15 pRep4 (Qiagen, Hilden, Germany) were used for *in vitro* RNA production, mutant construction, or recombinant protein synthesis, respectively. Media were supplemented with chloramphenicol (Chl; 10 µg/mL), kanamycin (Kan; 25 µg/mL), erythromycin (Em; 30 µg/mL) or ampicillin (Amp; 100 µg/mL) for plasmid selection as needed.

**TABLE 3:** Strains and plasmids used in this study

Strain or plasmid	Characteristics^*a*^	Reference
Strains		
*E. faecalis* V19	Van^R^; Δ*ABC*	(63)
*E. faecalis* V19 Δ*nagY*	Van^R^; Δ*ABC;* Δ*nagY*	(10)
*E. faecalis* V19 Δ5′ *nagY*	Van^R^; Δ*ABC;* Δ*5′ nagY*	(10)
*E. faecalis* V19 Δ*rnc*	Van^R^; Δ*ABC;* Δ*rnc*	(24)
*E. faecalis* V19 Δ*nagY*Δ*rnc*	Van^R^; Δ*ABC;* Δ*nagY*Δ*rnc*	This study
*E. faecalis* V19 Δ*nagE*	Van^R^*;* Δ*ABC;* Δ*nagE*	This study
*E. faecalis* V19 pUCB300-*nagY*-FLAG	Van^R^; Em^R^; Δ*ABC*; pUCB300 *nag*Y-FLAG	This study
*E. faecalis* V19 pIL252	Van^R^*;* Δ*ABC;* pIL252; Em^R^	This study
*E. faecalis* Δ*nagE* pIL252-*nagE*	Van^R^*;* Δ*ABC;* Δ*nagE* pIL252- *nagE* Em^R^	This study
*E. faecalis* Δ*nagY*Δ*rnc-rnc*-pIL252-*nagY*	Van^R^*;* Δ*ABC;* Δ*nagY*Δ*rnc* with chromosomal reintroduction of *rnc*; pIL252- *nagY;* Em^R^	This study
*E. coli* TOP10	*hsdR; mcrA; lacZ*Δ*M15; endA1; recA1*	Thermo Fisher
*E. coli* NEB-5α	*fhuA2*Δ(*argF-lacZ*)U169 *phoA glnV44*Φ80Δ(*lacZ*)M15 *gyrA96 recA1 relA1 endA1 thi-1 hsdR17*	New England BioLabs
*E. coli* DH5a	F^–^ φ80*lac*ZΔM15 Δ(*lac*ZYA-*arg*F)U169 *rec*A1 *end*A1 *hsd*R17(r_K_^–^, m_K_^+^) *pho*A *sup*E44 λ^–^*thi*-1 *gyr*A96 *rel*A1	Life Technologies
*E. coli* M15 pRep4	Amp^R^; Kan^R^; pRep4; *recA*	Qiagen
*E. coli* M15 pRep4 pQE70-*nagY*	Amp^R^; Kan^R^; pRep4; pQE70-*nagY*; *recA*	(10)
*E. coli* M15 pRep4 pQE30-*rnc*	Amp^R^; Kan^R^; pRep4; pQE30-*rnc*; *recA*	(24)
*L. lactis* MG1363	*repA33*	(65, 66)
Plasmids		
pQE70	6× His-tag C terminal; lac operator; ColE1 ori; Chl^R^; Amp^R^; MCS	Qiagen
pTOPO	*ccdB; lacZ-*a*;* pUC ori; Kan^R^; Zeo^R^; MCS	Thermo Fisher
pQE30	6× His-tag N terminal; lac operator; ColE1 ori; Chl^R^; Amp^R^; MCS	Qiagen
pLT06	*lacZ*; *repATS*; Chl^R^; MCS	(67)
pEBM2	*repA^TS^* ori; *lacZ-*α; P23-thyA*; *ori*T; Chl^R^; MCS	This study
pIL252	pAMβ1 derivative, Em^R^	(68)
pJH082	OG1Sp *upp4*::P23*repA4*	(69)
pCF10	67.7 Kb *E. faecalis* conjugative plasmid	(70)
pBluescript KS^+^	*E. coli* plasmid vector; Amp^R^	Stratagene
pUCB300	*lacZ*-α; pUC *ori*; Amp^R^; Ery^R^; MCS	(71)

^*a*^ Van, vancomycin; Zeo, zeomicin; ori, origin of replication; ts, thermosensitive; MCS, multiple cloning site.

### Streptozotocin inhibition assays

STZ inhibition assays were performed as previously described (72). After 16 h of incubation in GM17 supplemented with 0.5% NAG, the cells were centrifuged and the pellets were washed and diluted in fresh GM17 to an OD_600_ of 0.12. The cells were then incubated for 60 min to initiate growth and divided into three aliquots: the first one containing 50 µg.mL^−1^ STZ; the second containing 50 µg.mL^−1^ STZ + 0.5% NAG; and the last containing no supplement. The three cultures were incubated at 37°C, and growth was determined by monitoring OD_600_, using a microplate reader Model 680 (BioRad, Hercules, CA, USA).

### MICs

The MICs of STZ were determined by the broth microdilution technique, according to the Clinical and Laboratory Standards Institute guidelines, in GM17. Overnight cultures were diluted to an initial inoculum of 10^6^ CFU/mL. The MIC was defined as the lowest concentration that inhibited bacterial growth.

### Molecular biology techniques

The PCR reactions were performed using Q5 High-Fidelity DNA Polymerase (New England BioLabs) and GoTaq DNA Polymerase (Promega, Madison, WI, USA). Primers used in this study are listed in Table S6. NucleoSpin Gel and PCR Clean-up Kit (Macherey-Nagel, Düren, Germany) and NucleoSpin Plasmid Kit (Macherey-Nagel) were used for the purification of PCR products and plasmid extraction, respectively, according to the manufacturer’s recommendations. Digestion and ligation of genomic and plasmid DNA were carried out with restriction enzymes from Promega (Madison, WI, USA) or Thermo Fisher and T4 DNA Ligase from New England BioLabs, respectively.

### Construction of the pEBM2 vector

A new plasmid vector, pEBM2, was generated to facilitate mutagenesis by double crossing over. To construct this vector, we first amplified a 4.4 kb region containing the chloramphenicol resistance gene (*cat*), the P23-thyA* counterselection cassette, the origin of replication, and the *repATS* gene of the plasmid pJH082 using primers pLT06A and pLT06B (Table S6). On the other hand, a 117 bp long fragment, including the 40 bp corresponding to the minimal origin of transfer region (*ori*T) of plasmid pCF10 required for plasmid conjugation (73), was amplified from pCF10 using primers pCF10A and pCF10B. The two resulting DNA fragments were digested with the restriction endonucleases *Afl*II (Thermo Fisher) and *Apa*I (Promega) and then ligated with T4 DNA Ligase (Promega) according to the manufacturer’s recommendations. The pJH082oriT plasmid resulting from the fusion of these two amplimers was obtained after transformation of *E. coli* DH5α and selection on medium containing chloramphenicol at 30°C. Secondly, the genetic region of the gene encoding the α-subunit of LacZ and containing the multiple cloning site (MCS) of the pBluescriptSK^+^ vector was amplified using the pBSA and pBSB primers. The resulting 742 bp fragment generated was digested with the restriction endonucleases *Hpa*I and *Nhe*I (Promega) and cloned into pJH082oriT, which was previously digested with the enzymes *Sma*I and *Xba*I (Promega) to produce ends which are compatible with those resulting from restriction by *Hpa*I and *Nhe*I, respectively. The resulting plasmid (pEBM2) contains an MCS within the gene encoding the α-subunit of LacZ, allowing the screening of clones of interest by α-complementation. It replicates conditionally, thanks to the *repATS* gene, which encodes a temperature-sensitive replicase, making it easier to obtain clones resulting from an initial crossover event (74). The presence of the thyA* gene, which encodes a dominant-negative thymidylate synthase, significantly inhibits bacterial growth on a thymine-depleted medium, thereby facilitating the recovery of clones resulting from a second crossover event (69). In addition, pEBM2 also contains *ori*T, which can be used to transfer it by conjugation from an *E. faecalis* strain that also harbors pCF10 to other enterococci or to members of other bacterial genera (73).

### Construction of *E. faecalis* mutant strains

The ∆*nagE* (∆*ef1516*) and ∆*nagY*∆*rnc* (∆*ef1515*∆*ef3097*) mutant strains were constructed in *E. faecalis* V19 using *E. coli* NEB-5α as an intermediate cloning host (Table 3).

The ∆*nagY*∆*rnc* double mutant strain was constructed from the previously described ∆*rnc* mutant strains (24) using the *in vivo* recombination method with the pLT06 vector (Chl^R^) as previously described before for the construction of the ∆*nagY* mutant (10, 67, 75). To construct ∆*nagE* strain, flanking regions of the chromosome region to be deleted were amplified by PCR using oligonucleotides ef1516_1_IVR and ef1516_2_IVR for the upstream fragment, and oligonucleotides ef1516_3_IVR and ef1516_4_IVR for downstream fragment (Table S6). A nested PCR was performed with oligonucleotides ef1516_1_*Bam*HI and ef1516_4_*Eco*RV carrying restriction enzyme sites (Table S6). This PCR fragment and the pEBM2 vector (Chl^R^) were digested with *Bam*HI and *Eco*RV, ligated together, and transformed into *E. coli*. The deletion was obtained by double crossover in *E. faecalis* V19, as previously described (67), and verified by PCR using primers ef1516_5 and ef1516_6 (Table S6) and by sequencing (Eurofins Genomics, Cologne, Germany).

The *in vivo* recombination method was also used to construct the *E. faecalis* V19 strain containing the chromosome-integrated pUCB300-*nagY*-FLAG (Table 3) (75). The pUCB300 vector (Amp^R^; Ery^R^) (71) and the *nagY* gene were amplified using primers pUCB300_rec_rev/pUCB300_rec_for and pUCB300_FLAG_1515F/pUCB300_1515R, respectively (Table S6). Primer pUCB300_FLAG_1515F was constructed by integrating the FLAG tag sequence (CTTGTCATCGTCGTCCTTGTAGTC). *E. coli* strain TOP10 was used as an intermediate cloning host (Table 3), and plasmid construction was verified by PCR with primers pUCB300/pUCB300R followed by sequencing (Eurofins Genomics) (Table S6). Integration of the plasmid into the chromosome of *E. faecalis* V19, which occurs immediately after electroporation since it was not replicative in these bacteria, was verified by PCR with a plasmid primer and a chromosomal primer (upstream insertion: ef1516_5/pUCB300F; downstream insertion: pUCB300R/ef1515_6).

### Construction of the complemented strains

To complement the Δ*nagY*Δ*rnc* double mutants, the *rnc* gene was reintroduced on the genome using the pMAD vector (68), as described for the Δ*rnc* mutant construction, by using the primers rnc-compF and rnc-compR to amplify the gene (24). To complement the Δ*nagE* and Δ*nagY* deletions in *trans*, the *nagE* and *nagY* genes were amplified by PCR from V19 chromosomal DNA with primers nagE or nagY-compF and compR, respectively (Table S6). The low copy number gram-positive plasmid pIL252 derivative (76) was linearized by the restriction enzyme *Sac*I (Promega). Assembly of the DNA molecules was performed using the NEBuilder Hifi DNA Assembly Master Mix (NEB), yielding the plasmid pIL252-*nagE* or *nagY* after electroporation in *Lactococcus lactis* MG1363*repA*33. The plasmid sequences were confirmed by sequencing and then introduced into the corresponding mutants to construct the complemented strain (Table 3). Control strains containing the empty pIL252 plasmid were also constructed (V19 + pIL252).

### Total RNA extraction

Cultures for RNA extraction intended for qPCR or RNA-Seq were prepared in carbon-depleted medium cdM17 (77) supplemented with the appropriate sugar, or in GM17, and incubated at 37°C until OD_600_ 0.5. One-hour induction in cdM17, GM17, or M17 with 0.5% NAG was performed after two cell washes in physiological water (NaCl 0.9%).

Cells were pelleted and resuspended in 500 µL TE buffer (pH 8; 10 mM Tris; 0.1 mM Na_2_EDTA), then lysed in the FastPrep device (2 × 30 s at four movements/s; MP Biomedicals, Illkirch Graffenstaden, France). TRIzol reagent (Thermo Fisher) was added to the lysates, incubated for 5 min at room temperature, and centrifuged at 11,600 × *g* for 2 min. The supernatant was mixed with 1 vol of isoamyl alcohol-chloroform, centrifuged, and transferred to a new tube containing 1 vol of absolute ethanol. RNA was purified using the Direct-Zol RNA Miniprep Kit (Zymo-Research, Irvine, CA, USA) according to the manufacturer’s recommendations and quantified using Nanodrop 2000 (Thermo Fisher). RNA quality was checked by 1% agarose gel electrophoresis.

### RNA expression and transcriptional start site identification

RNA was reverse transcribed into cDNA using the QuantiTect Reverse Transcription Kit (Qiagen) according to the manufacturer’s recommendations. The qPCR was then performed with L/R oligonucleotides for RT-qPCR (Table S6) using the GoTaq qPCR Master Mix kit (Promega) and a C1000 Thermal Cycler (Bio-Rad, Hercules, CA, USA), with the following program: 3 min at 95°C, 40 cycles of 15 s at 95°C, and 1 min at 60°C. The *gyrA* DNA gyrase subunit A gene was used to normalize the transcript levels of each gene tested. The qPCR efficiency was determined using standard curves generated with genomic DNA from the *E. faecalis* V19 reference strain.

5′ RACE experiments were performed with the 5′/3′ RACE kit, 2nd generation (Roche, Bale, Switzerland), according to the manufacturer’s recommendations, using EF1515R as SP1 (RT), ef1515_SP2 (PCR with anchor primers), and ef1515_SP3 (sequencing) primers (Table S6) and poly-C tailing.

### *In vitro* production of RNA

*In vitro* expression of 5′ *nagY* was performed as previously described using the MAXIScript T7 or the MEGAScript T7 Transcription Kits (Invitrogen, Carlsbad, CA, USA), followed by two consecutive DNase treatments, i.e., DNase from the transcription kit and the TURBO DNase (Invitrogen), as recommended by the supplier. RNA was then purified by an ammonium acetate/ethanol precipitation, resuspended in RNase-free water, and quantified by the Nanodrop 2000 (Thermo Fisher) or by OD_260_. The absence of DNA contamination was verified by RT-PCR using the QuantiTect Reverse Transcription Kit (Qiagen), with negative control without retrotranscriptase, and GoTaq DNA Polymerase (Promega), with TOPO85_RP1 and TOPO85_FP1 primers for PCR (Table S6), followed by an agarose gel electrophoresis.

### Synthesis and purification of recombinant proteins

The RNase III and NagY proteins were produced from *E. coli* M15 pRep4 strains containing pQE30-*rnc* and pQE70-*nagY* vectors, respectively (10, 24). Cultures of *E. coli* M15 pRep4 pQE30-*rnc* and *E. coli* M15 pRep4 pQE70-*nagY* were prepared in Terrific Broth medium, supplemented with Kan and Amp and incubated at 37°C with shaking until OD_600_ 0.5. Expression induction was triggered with 0.5 mM isopropyl-β-D-1-thiogalactopyranoside for 3 h at 37°C under agitation. Cells were pelleted, resuspended in buffer A (pH 7.5; NaCl 0.5 M; Tris/HCl 25 mM), washed, and resuspended in lysis buffer B (pH 7.5; NaCl 0.5 M; Tris/HCl 25 mM; imidazole 10 mM) supplemented with 1 mg/mL lysozyme. After a 30 min incubation at 4°C under agitation by inversion, bacterial lysis was completed on the Fisherbrand Model 120 Sonic Dismembrator (Fisher Scientific, Hampton, NH, USA) with the following parameters: 2 min 30, pulse mode 15 s/15 s, amplitude 50%, and then the soluble fraction was collected. RNase III and NagY proteins were purified using the ÄKTA Start protein purification system and HisTrap HP His-tag protein purification columns according to the manufacturer’s recommendations (Cytiva, Marlborough, MA, USA). During purification, buffers B and C (pH 7.5; 0.5 M NaCl; 25 mM Tris/HCl; 500 mM imidazole) were used to achieve linear elution from 15 mM to 500 mM imidazole, and EB buffer (pH 7.5; 0.5 M NaCl; 25 mM Tris/HCl; 10% glycerol) was used to elute proteins. Purified proteins were then concentrated by ultrafiltration on a Vivaspin 30 kDa membrane (Sigma-Aldrich, Saint-Louis, MO, USA) and desalted on a PD10 column (Cytiva) previously equilibrated with EB buffer. A 12.5% SDS-PAGE was performed to check the purity of the proteins and a bicinchoninic acid (BCA) assay (Thermo Fisher) to determine their concentration.

### Spectral shift

The interaction between RNase III, NagY, and/or 5′ *nagY* was determined in a non-cellular manner by fluorescence spectral shift using a Monolith X automated system (Nanotemper Technologies, Munich, Germany). Recombinant RNase III protein was Cys-labeled under reductive conditions using the Protein Labeling Kit RED-MALEIMIDE 2nd Generation (Nanotemper Technologies, München, Germany) according to the manufacturer’s recommendations. Briefly, protein (100 µL) was prepared at a concentration of 10 µM in ES-T buffer (pH 8.0; 10 mM Tris, 40 mM NaCl, 10 mM KCl, 1 mM MgCl_2_, 0.05% Tween-80) and then labeled by adding 10 µL of a 265 µM solution of Red-Maleimide 2nd Generation dye (10:1 mol:mol ratio), in the presence of 5 mM tris(2-carboxyethyl)phosphine as a reducing agent for 30 min in the dark. The labeled protein (RNase III*) was then purified from the remaining free dye by gravity gel filtration using the column provided by the manufacturer. The final concentration and DOL of RNase III* was quantified using a Nanodrop 2000 (Thermo Fisher) UV-visible spectrophotometer, and the stock solution was diluted in ES-T buffer to a final concentration of 5·10^−8^ M. NagY protein and 5′ *nagY* RNA, which has been heated at 70°C for 5 min and then slowly cooled to room temperature to allow proper formation of secondary structures, were also diluted in ES-T buffer at various concentrations.

A pre-test was performed to check the fluorescence intensity and the absence of adsorption or aggregation of the labeled and diluted molecule. Various assays were then carried out to investigate the interaction between (Table S1) (i) RNase III* and 5*′ nagY,* serially diluted to obtain a final concentration ranging from 1.3·10^−9^ to 4.3·10^−5^ M, (ii) RNase III* and NagY from 5.2·10^−10^ to 1.7·10^−5^ M, and (iii–v) three-partner interaction with RNase III* and NagY at a final concentration of 8.5·10^−6^ M and 5′ *nagY* serially diluted from 2.6·10^−10^ to 8.5·10^−6^ M for test 3, from 1.6·10^−10^ to 5.3·10^−6^ for test 4, and from 1.3·10^−9^ to 4.3·10^−5^ M for test 5. The different tests were performed by incubating the first two partners for 5 min at room temperature before adding the third partner. Specifically, test C was performed by adding NagY and 5′ *nagY* followed by RNase III*, the test D by adding RNase III* and NagY followed by the 5′ *nagY*, and the test E by adding RNase III* and 5′ *nagY*, followed by NagY. While each concentration of RNA or NagY was mixed volume to volume (1:1) with the labeled protein for binary experiments, the two proteins (NagY or RNase III*) and the RNA were added to give a final ratio of 0.5:0.5:1 when there were three partners. The set was then filled into 10 µL capillaries, introduced into the Monolith X instrument (NanoTemper Technologies), and performed in independent triplicates (MO.Control software version 2.6.3, NanoTemper Technologies).

### Co-immunoprecipitation assay

The co-immunoprecipitation was performed as published by Lioliou et al. with the modification described below (26). The target RNA of the NagY protein was obtained from *E. faecalis* V19 and V19 pUCB300-*nagY*-FLAG strains, thanks to the FLAG tag carried by NagY. Cultures from these strains were first diluted 1:100 in 100 mL GM17 and incubated at 37°C until OD_600_ 0.5. Cells in the exponential growth phase were centrifuged at 4,500 × *g* for 10 min, and the pellets were washed twice with physiological water (NaCl 0.9%). Bacteria were then incubated in M17 supplemented with NAG 0.5% for 1 h at 37°C, centrifuged as above, and then resuspended in 2 mL buffer (pH 8; 50 mM Tris; 150 mM NaCl; 1 mM EDTA; 1% Triton; Mini cOmplete protease inhibitor [Roche]). This chemical and enzymatic lysis was followed by mechanical lysis using the FastPrep (2 × 40 s at 4.0 movements/s) (MP Biomedicals). After centrifugation at 33,000 × *g* for 15 min at 4°C, the supernatant was collected and protein concentration was determined using Pierce BCA Protein Assay Kits (Thermo Fisher), following the supplier’s recommendations. Immunoprecipitation was performed by adding 2 mL of lysates to 160 µL of anti-Flag-M2 affinity agarose beads (80 µL of packed gel volume) (Sigma-Aldrich), previously washed two times in Tris-buffered saline (TBS) (pH 7.4; 10 mM Tris HCl; 150 mM NaCl), for 2 h at 4°C with wheel agitation. The samples were then washed three times with TBS and centrifuged at 5,000 × *g* for 1 min. Finally, elution was performed with FLAG peptide (Merck, Darmstadt, Germany) according to the supplier’s recommendations. FLAG peptides were co-incubated with beads for 30 min at 4°C with agitation, at a final concentration of 100 µg/mL in TBS. After centrifugation, the supernatant was collected, and RNAs were extracted with TRIzol reagent (Thermo Fisher) and isoamyl alcohol-chloroform (24:1), as described above (section Total RNA extraction). Finally, the RNAs were resuspended in 50 µL of RNase-free water, quantified using Nanodrop 2000 (Thermo Fisher), and sent for sequencing.

### Sequencing analysis

mRNA fragmentation, strand-specific cDNA synthesis, ribodepletion, library construction, and Illumina paired-end sequencing were subcontracted to Eurofins Genomics.

Mapping, normalization, and read counting were carried out on the reference genome AE016830.1 retrieved from the NCBI server, using CLC Genomics Workbench (12.0.2). RNA-Seq data are available in NCBI’s SRA database (PRJNA1221897), and reports are presented in Table S2. The data were then normalized to a global fit and sorted according to their TPM (transcripts per kilobase million). The TPMs of *E. faecalis* WT, Δ*nagY*, ∆*rnc,* or ∆*nagY*∆*rnc* strains were compared using the ratio TPM mutant/TPM WT. Genes with a ratio of less than or greater than five were selected, and gene function was determined using NCBI Gene tools (https://www.ncbi.nlm.nih.gov/gene/) and the UniProt database (https://www.uniprot.org/).

NagY co-immunoprecipitated RNAs were analyzed as described by Lioliou et al. (26) using CLC Genomics Workbench (12.0.2), with data accessible in NCBI’s SRA database (PRJNA1279004). The resulting mappings were visualized using the Integrated Genome Browser to allow the precise location of reads, to take into account their position within a UTR, as is the case for transcriptional antiterminators (Table S5). UTRs were manually determined using the transcriptome map available at http://www.helmholtz-hiri.de/en/datasets/enterococcus (30) and whole-genome mapping of 5′ RNA from previous works (17, 31).

### Bioinformatics and statistical analyses

Putative RAT sequences are identified using the FIMO (Find Individual Motif Occurrences) tool, with the 5′ *nagY* RAT sequence “TTTCATGTGACTAGATAATACTAGGCATGGAA” as reference, searching for occurrences with *P*-values of less than 0.01 (32).

The data obtained from at least three independent experiments were subjected to one-way analysis of variance by using Prism 6 software (GraphPad software, San Diego, USA). Tukey’s test was selected to determine the significant differences between the variables.

## SUPPLEMENTAL MATERIAL

Supplemental material spectrum.01137-25-s0001.docx Methods; Tables S1 and S2, S4, S6; Fig. S1 to S4.

Table S3 spectrum.01137-25-s0002.xlsx Results of RNA-Seq assays.

Table S5 spectrum.01137-25-s0003.xlsx Genes selected after RIP-Seq assay.

ASM does not own the copyrights to Supplemental Material that may be linked to, or accessed through, an article. The authors have granted ASM a non-exclusive, world-wide license to publish the Supplemental Material files. Please contact the corresponding author directly for reuse.
