# Supplementary material for: The NagY antiterminator in Enterococcus faecalis: a novel regulatory mechanism and its impact on cell metabolism
Source: Microbiol Spectr. 2025 Aug 29;13(10):e01137-25. doi: 10.1128/spectrum.01137-25 (PMC12502629; doi:10.1128/spectrum.01137-25)
Supplement: Supplemental material — Methods; Tables S1 and S2, S4, S6; Fig. S1 to S4. [file spectrum.01137-25-s0001.docx]

**Supplementary method**

***Western blotting***

The V19 pUCB300-*nagY*-FLAG strain was cultivated as described in the “co-immuniprecipitation assay” described in the main text. Bacteria were then harvested and lysed at 4 time points (0, 15 min, 30 min and 1h). Western blot analysis was then performed to verify the expression of NagY-FLAG. Fifty μg of the lysate were deposited in 12.5 % SDS-PAGE gel; with PageRuler™ 10 at 180 kDa size marker (ThermoFisher). Two gels were prepared, one of which was stained with Coomassie Blue and the second transferred onto a 0.45 μm Immobilon-P PVDF (polyvinylidene fluoride) membrane (Merck, Darmstadt, Germany), using the Pierce™ Power Blotter system (ThermoFisher) with the following parameters: 10 min transfer; 1 gel; high. The PVDF membrane was first rehydrated in methanol until transparent and then equilibrated in transfer buffer (12 mM Tris-HCl pH8.3;96 mM glycine, 20% methanol (v:v)) for 20 min. After protein transfer, the membrane was washed and incubated in blocking solution (1% BSA (w/v) in TBS) 1 h at room temperature with stirring. The membrane was then washed twice in TBS. Primary antibody detection was performed with the mouse monoclonal anti-FLAG® M2 (Sigma Aldrich) at 2 x 10^-3^ mg/mL in TBS and incubated for 3h at room temperature with agitation. After 3 min of rinsing in TBS, the PVDF membrane was incubated for with the secondary antibody anti-mouse IgG peroxidase (Sigma-Aldrich) at 2 x 10^-4^ mg/mL in blocking solution for2 h at room temperature. The membrane was washed again three times for 5 min in TBS. Finally, the Clarity Max Western ECL Blotting Substrate kit (Bio-Rad) was used, according to the supplier's recommendations, and the signal was acquired using the ChemiDoc Imaging System (Bio-Rad).

The second gel was stained with Coomassie Blue for 1 h, then destained twice in 10 % acetic acid/45 % methanol for 30 min and 3h.

**Supplementary Table S1:** Summary of microscale thermophoresis experiments.

| Assay | 1 | 2 | 3 | 4 | 5 |
| --- | --- | --- | --- | --- | --- |
| Labeled target^a^ | RNase III 5·10^-8^M | RNase III  5·10^-8^M | RNase III  8.5·10^-6^M | RNase III  8.5·10^-6^M | RNase III  8.5·10^-6^M |
| Ligand^b^ | 5’*nagY* | NagY | 5’*nagY* | 5’*nagY* | 5’*nagY* |
| Ligand concentration range | 1.3·10^-9^ - 4.3·10^-5^M | 5.2·10^-10^ –  1.7·10^-5^M | 1.6·10^-10^ –  5.3·10^-6^M | 2.6·10^-10^ –  8.5·10^-6^M | 1.3·10^-9^ –  4.3·10^-5^M |
| Experiments^c^ | Binary | Binary | Ternary with NagY (8.5·10^-6^ M) | Ternary with NagY (8.5·10^-6^ M) | Ternary with NagY (8.5·10^-6^ M) |
| Pre-incubation^c^ | None | None | NagY + 5’*nagY* | NagY + RNase III | RNase III + 5’*nagY* |
| Kd | / | 2.5 ·10^-6^ M  (CI: 0.9 - 7·10^-6^ M) | / | 5.8 10^-7^ M  (CI: 2.4 – 13.8 10^-7^ M) | / |
| Related figure | Figure S1  Panel A | Figure 4  Panel A | Figure S1  Panel B | Figure 4  Panel B | Figure S1  Panel C |

^a^ the target refers to the labeled protein. ^b^ the ligand refers to the component added in variable amounts through serial dilution. ^c^ for ternary experiments, two partners were incubated first, followed by the addition of the third one 5 min later. CI: confidence interval

**Supplementary Table S2:** Read numbers and mapping statistics of RNA-Seq assays

| **Features** | **V19 (WT)** | **Δ*nagY*** | **Δ*rnc*** | **Δ*nagY* Δ*rnc*** |
| --- | --- | --- | --- | --- |
| **Total number of reads** | 31,153,452 | 28,882,474 | 19,337,658 | 33,651,502 |
| **Paired, mapped pairs %** | 90.69 | 84.14 | 92.61 | 95.47 |
| **Paired, broken pairs %** | 7.45 | 12.96 | 5.87 | 2.26 |
| **Paired, not mapped %** | 1.86 | 2.90 | 1.52 | 2.27 |
| **Mapped to genes %** | 89.23 | 79.27 | 81.11 | 92.29 |
| **Ignored reads %** | 0 | 0 | 0 | 0 |
| **Number of reads for mRNA %** | 88.91 | 88.13 | 89.22 | 88.68 |
| **Numbers of reads for ncRNAs and tmRNA%** | 4.23 | 3.26 | 8.5 | 3.48 |
| **Numbers of reads for tRNA %** | 0 | 0.02 | 0 | 0 |

**Supplementary Table S3 (.xlsx):** Results of RNA-Seq assays. RNAs were prepared from the 4 indicated strains, in exponential growth phase followed by 1 h in the presence of N-acetylglucosamine. TPM: Transcripts Per kilobase Million

**Supplementary Table S4:** Read numbers and mapping statistics of RIP-Seq assays

| **Features** | **V19**  **(WT untagged)** | **V19 NagY-FLAG** |
| --- | --- | --- |
| **Total number of reads** | 33,996 | 32,544 |
| **Mapped reads % (inside UTR)** | 78.96 (23%) | 79.23 (65%) |
| **Reads >18nt %** | 60.81 | 77.96 |
| **Total number of mappings** | 4755 | 1755 |

**Supplementary Table S5 (.xlsx):** Genes selected after RIP-Seq assay corresponding to mRNAs with at least 5 reads co-precipitated with NagY-FLAG (from "raw data" sheet). The positions of interest (UTR or RAT position in the UTR) and reads location after co-IP are indicated, from the ATG of the identified open reading frame (accessible at https://bacteroides.helmholtz-hzi.de/enterococcus/, and in Innocenti et al., 2015 or Muller et al., 2015). Additional information is provided in the "comments" column. In green: NagY target genes with repressed expression in the Δ*nagY* mutant compared to the wild-type strain, in gray: RNase III known targets. ND: not determined.

**Supplementary Table S6:** List of primers and their corresponding nucleotide sequences used in this study.

| **Name** | **Sequence ^a^** | **Use** |
| --- | --- | --- |
| ef1516_1_IVR | GTACCCGGGGATCCTCTAGAGTTAGAATAAGCACTGTTAACA | *ef1516* mutant |
| ef1516_2_IVR | CATTTCCGATTACAATTAACGAACGTCCCATTC | *ef1516* mutant |
| ef1516_3_IVR | GGGACGTTCGTTAATTGTAATCGGAAATGTTACCG | *ef1516* mutant |
| ef1516_4_IVR | CGCTGGGTTTATCGACCTGCAGATTTTGCTACATTTTCCTCAT | *ef1516* mutant |
| ef1516_1_*Bam*HI | atatggatccATACAATGTGGTGTACAATG (*Bam*HI) | *ef1516* mutant |
| ef1516_4_*Eco*RV | TTATGATATCCAGGTCGACTTTATTCAACA (*Eco*RV) | *ef1516* mutant |
| ef1516_5 | AAAAGTATTGGTGGAAGAAGAT | *ef1516* mutant and pUCB300-*nagY*-FLAG strain |
| ef1516_6 | CATTTCGAACAACATGAACAT | *ef1516* mutant and pUCB300-*nagY*-FLAG strain |
| 3097-5 | TTCTGTTCGTTCATATTCTTTTG | *ef1515 ef3097* mutant |
| 3097-6 | TTTTTCTGCGATTGCTGTTTC | *ef151 5ef3097* mutant |
| SRC_85_5 | AAACGAAGCATTCGTGAAAGA | *ef1515 ef3097* mutant |
| ef1515_6 | CCAACATCATTGGATGATTCG | *ef1515 ef3097* mutant |
| nagY-compF | CAGCTTATCATCGGAGCTCCATGAAAATTAAAAAGGTGCT | Complementation of *nagY* deletion |
| nagY-compR | ACGGATCCCCTACTAAAACGTAACTCCTCACATAAACT | Complementation of *nagY* deletion |
| nagE-compF | CAGCTTATCATCGGAGCTCCATGAAAGCGTATATGCAAAG | Complementation of *nagE* deletion |
| nagE-compR | ACGGATCCCCTACTAAAATGGCTCTACAGACACTAATA | Complementation of *nagE* deletion |
| rnc-compF | TAGTGGATCCAATACCGTTGTTAAAATATT | Complementation of *rnc* deletion |
| rnc-compR | TGTTTCCCGGGTCTGCAATTCTTCTCGA | Complementation of *rnc* deletion |
| pUCB300_**FLAG**_1515F | GGTAACGCCAGGGTTTTCCCAGTCACGACGCTA**CTTGTCATCGTCGTCCTTGTAGTC**TTTGGTCATTCTTAATCGTT | pUCB300-*nagY*-FLAG strain |
| pUCB300_1515R | TAACAATTTCACACAGGAAACAGCTATGACATGAAAATTAAAAAGGTGCT | pUCB300-*nagY*-FLAG strain |
| pUCB300R | CGTCGTGACTGGGAAAACC | pUCB300-*nagY*-FLAG strain |
| pUCB300F | GTCATAGCTGTTTCCTGTGTG | pUCB300-*nagY*-FLAG strain |
| pLT06A | attcTTAAgGTCAAATGGTACTGATGACC (*Afl*II) | pEMB2 construction |
| pLT06B | tatgggCccAAAACAAGTTAAGGGATGCAG (*Apa*I) | pEMB2 construction |
| pCF10A | tatgggCCcACCAGTATTTTTGGTAGGGG (*Apa*I) | pEMB2 construction |
| pCF10B | TTACtTAAgAAGTAGTCTCCTTAGTTTGAC (*Afl*II) | pEMB2 construction |
| pBSA | TtAtgttAACGCAATTAATGTGAGTTAG (*Hpa*I) | pEMB2 construction |
| pBSB | tTatgctaGCGAAAAACCGTCTATCAG (*Nhe*I) | pEMB2 construction |
| pIL252L | GGAGCTCCGATGATAAGCTG | pIL252 amplification |
| pIL252R | TTTTAGTAGGGGATCCGTCGACCTG | pIL252 amplification |
| ef1515L | AGTAGCTGTCGGTAAAGGCG | qPCR |
| ef1515R | AGTCCTTCTGGCTCCATCAC | qPCR and RACE-PCR |
| ef1516L | ATGTGCACGTCCTGAGAAGA | qPCR |
| ef1516R | GCCAGCTGTCCAATGCATAA | qPCR |
| ef0020L | ACGGTGAATTTGCTGAAGGGA | qPCR |
| ef0020R | CATCATCGGGACCTTCGCTA | qPCR |
| gyrAL | GATGGGGAAATCAGGGATTC | qPCR |
| gyrAR | TCTTTTCCATTCGGCATTTC | qPCR |
| ef1515_SP2 | TATTAAACCCGACGCCTTTAC | RACE-PCR |
| ef1515_SP3 | TTCTCCTGTCCTTCGTCAAG | RACE-PCR |
| TOPO85_FP1 | taatacgactcactataGTTAACAAATGAATAGCGTTTTC | RT-PCR |
| TOPO85_RP1 | agtgtgctggaattcATGACCCTCTTCTGCAGAA | RT-PCR |

*^a^* Underlined bases correspond to restriction sites (between brackets). Bold bases correspond to the FLAG tag sequence.


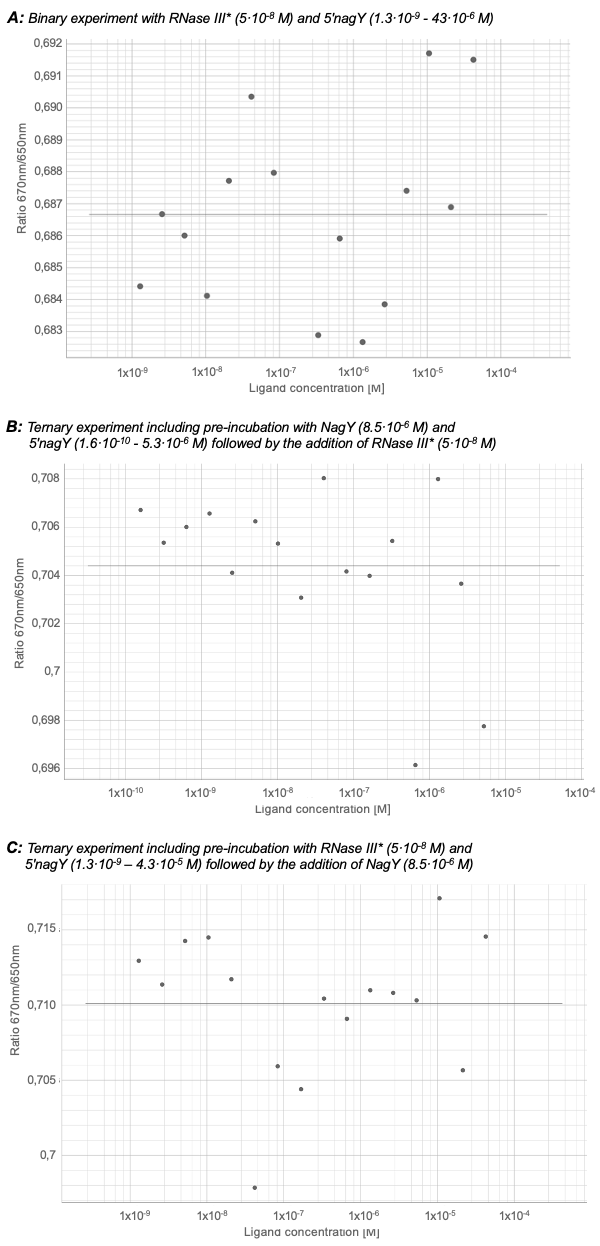


**Supplementary Figure S1:** Investigation of the interaction of RNase III-labeled (RNase III*) interaction with NagY and/or its 5'UTR. Spectral shifts (OD 670_nm_/650_nm_) are shown for the interaction between: (A) RNase III* (5·10^-8^ M) and 5'*nagY* (1.3·10^-9^ - 43·10^-6^ M), (B) NagY (8.5·10^-6^ M) and 5'*nagY* (1.6·10^-10^ - 5.3·10^-6^ M) followed by the addition of RNase III* (5·10^-8^ M), then (C) RNase III* (5·10^-8^ M) and 5'*nagY* (1.3·10^-9^ – 4.3·10^-5^ M) followed by the addition of NagY (8.5·10^-6^ M). Figures A, B and C refer to assays 1, 3 and 5; respectively: see Table S1 for more details.


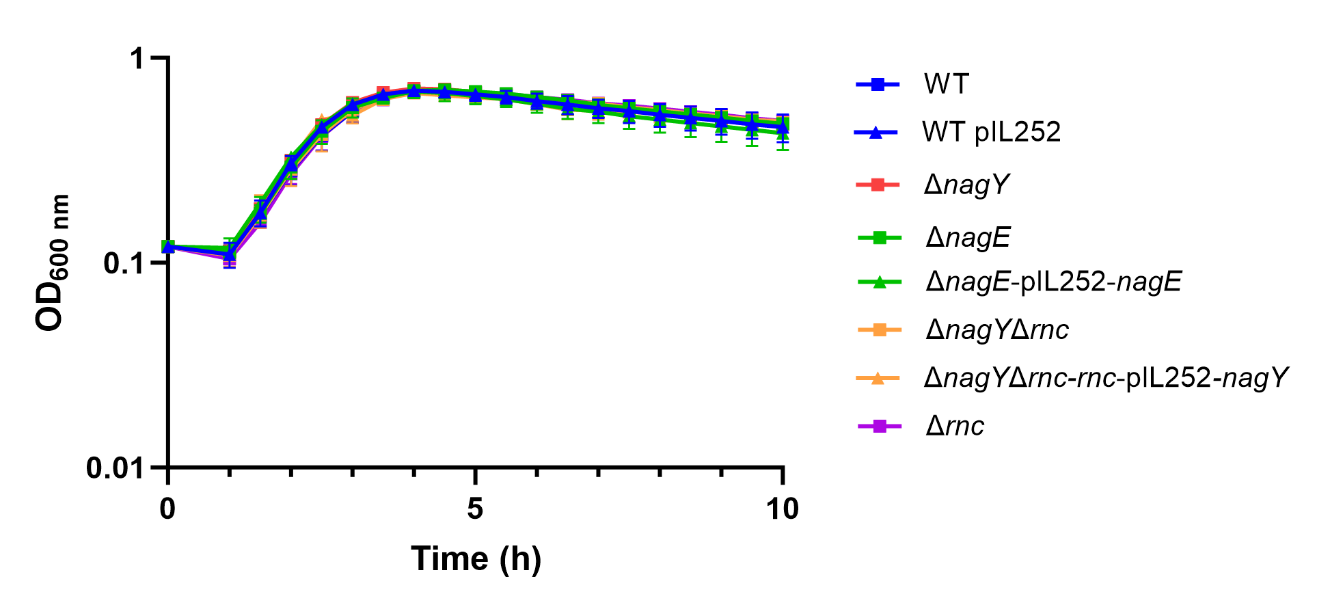


**Supplementary Figure S2:** Growth in the absence of streptozotocin (STZ). *E. faecalis* strains growths were monitored in GM17. Overnight cultures were performed in GM17 without NAG. Error bars represent data from three independent experiments, and data were analyzed with the Tukey multiple comparison test.


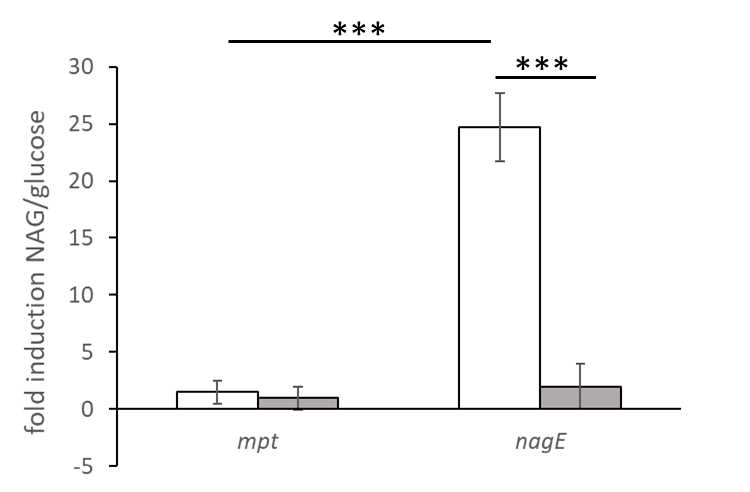


**Supplementary Figure S3:** Fold-change of *mptB* and *nagE* gene expression in the presence of NAG compared to glucose condition. RT-qPCR was performed on RNA extracted from *E. faecalis* V19 (WT, in white), and ∆*nagY* strain (in grey), in exponential phase and then exposed to glucose or NAG as sole carbon source for 1 h, in cdM17. Error bars represent data from triplicate independent experiments. Statistically significant differences were determined by the Tukey multiple comparison test, ***p<0.0001


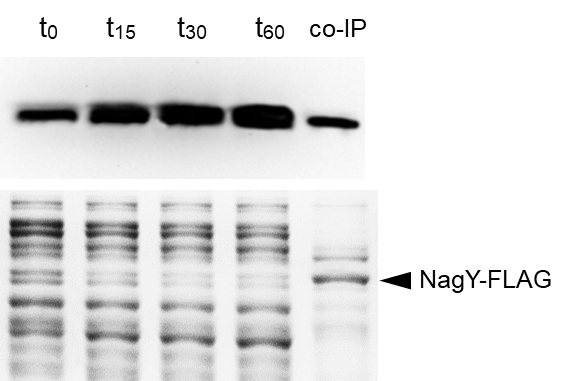


**Supplementary Figure S4:** NagY-FLAG stability. NagY-FLAG protein levels at mid-logarithmic phase were monitored at 4 time points of incubation with NAG (0, 15 min, 30 min and 1h) (top panel). Western blot was performed with a mouse anti-FLAG monoclonal antibody followed by an anti-mouse IgG peroxidase secondary antibody as described in the Supplementary Method section. A Coomassie-stained portion of the gel is shown as a loading control (lower panel).
